# Current and Future Role of Medical Imaging in Guiding the Management of Patients With Relapsed and Refractory Non-Hodgkin Lymphoma Treated With CAR T-Cell Therapy

**DOI:** 10.3389/fonc.2021.664688

**Published:** 2021-05-28

**Authors:** Laetitia Vercellino, Dorine de Jong, Roberta di Blasi, Salim Kanoun, Ran Reshef, Lawrence H. Schwartz, Laurent Dercle

**Affiliations:** ^1^ Nuclear Medicine Department Saint Louis Hospital, Assistance Publique Hôpitaux de Paris, Paris, France; ^2^ Center for Cell Engineering, Memorial Sloan Kettering Cancer Center, New York, NY, United States; ^3^ Onco-Hematology Department Saint Louis Hospital, Assistance Publique Hôpitaux de Paris, Paris, France; ^4^ Cancer Research Center of Toulouse (CRCT), Team 9, INSERM UMR 1037, Toulouse, France; ^5^ Blood and Marrow Transplantation and Cell Therapy Program, Division of Hematology/Oncology and Columbia Center for Translational Immunology, Columbia University Irving Medical Center, New York City, NY, United States; ^6^ Department of Radiology, New York Presbyterian, Columbia University Irving Medical Center, New York City, NY, United States

**Keywords:** lymphoma, CAR T-cell, immunotherapy, FDG PET/CT, CT scan, prognostic biomarker

## Abstract

Chimeric antigen receptor (CAR) T-cells are a novel immunotherapy available for patients with refractory/relapsed non-Hodgkin lymphoma. In this indication, clinical trials have demonstrated that CAR T-cells achieve high rates of response, complete response, and long-term response (up to 80%, 60%, and 40%, respectively). Nonetheless, the majority of patients ultimately relapsed. This review provides an overview about the current and future role of medical imaging in guiding the management of non-Hodgkin lymphoma patients treated with CAR T-cells. It discusses the value of predictive and prognostic biomarkers to better stratify the risk of relapse, and provide a patient-tailored therapeutic strategy. At baseline, high tumor volume (assessed on CT-scan or on [18F]-FDG PET/CT) is a prognostic factor associated with treatment failure. Response assessment has not been studied extensively yet. Available data suggests that current response assessment developed on CT-scan or on [18F]-FDG PET/CT for cytotoxic systemic therapies remains relevant to estimate lymphoma response to CAR T-cell therapy. Nonetheless, atypical patterns of response and progression have been observed and should be further analyzed. The potential advantages as well as limitations of artificial intelligence and radiomics as tools providing high throughput quantitative imaging features is described.

## Introduction

In 2017, reprogramming T lymphocytes to carry chimeric antigen receptor (CAR) targeting CD19 antigen became a novel immunotherapy commercially available for patients with refractory/relapsed B cell malignancies. Despite the unprecedented therapeutic responses achieved by CD19-CAR T-cells, the number of patients experiencing relapse stresses the need for reliable biomarkers to closely monitor clinical response and implement early consolidation strategies. Medical imaging such as [18F]-FDG PET/CT is already used for diagnosis and evaluation of hematologic malignancies and the clinical significance of several PET parameters such as Total Metabolic Tumor Volume, standardized uptake value and Total Lesion Glycolysis consumption has been extensively demonstrated. Hence, this is an area of ongoing investigation in the context of CAR T-cell therapy. This review aims to summarize recent clinical data and to emphasize the importance of further investigation of medical imaging biomarkers for CAR T-cells to optimize and personalize medical care: risk stratification, prediction of response, response assessment, and early detection of relapse.

## CAR T-Cell Manufacturing and Treatment

CAR T-cell manufacturing begins with T-cell collection from patients or donors by aphaeresis. These cells are then genetically reprogrammed (e.g., using viral vectors) to express receptors for specific tumor antigens. CD19 CAR T-cell therapy uses a single chain variable fragment (scFv) derived from the variable heavy and variable light chains of an antibody against epitopes of the CD19 antigen. In second generation CARs, the scFv is connected through a transmembrane domain to a costimulatory domain (such as CD28 or 4-1BB) further linked to the CD3ζ intracellular signaling domain of the T-cell receptor (1, 2). Before CAR T-cell infusion, patients are often given a bridging therapy to control their disease during the CAR T-cell production which may take two to six weeks. Patients then receive a lymphodepleting treatment several days before infusion to create a favorable environment for CAR T-cells by removing unmodified T-cells and immunosuppressive regulatory T-cell and increase homeostatic cytokine levels (3). Finally, cells are expanded and infused back into patients to achieve tumor cell recognition and killing. The antitumor response driven by CAR T-cells is HLA independent and relies on antigen-receptor binding and on the co-stimulatory signals that enhance T-cell proliferation and/or persistence. After antigen recognition, CAR T-cells eliminate cancer cells through death receptors-, cytokines- or granzyme/perforin-induced killing.

## Current Clinical Trial Landscape

As CAR-T-cells are still entering routine clinical practice, most of the current knowledge about imaging and outcomes has been gained from review of clinical trials. The most common cancer subtypes studied are acute lymphoblastic leukemia and non-Hodgkin lymphoma and the majority of studies used an autologous cell source (4). While the treatment of relapsed/refractory leukemia and lymphoma is increasing in clinical practice, and the first CAR T product for multiple myeloma was just recently FDA-approved (5, 6), the applications of this therapy in solid cancers remain at a nascent stage and need further investigations (7). Of note, medical imaging guides the management of lymphoma patients while it has a more limited impact for leukemia. Therefore, the focus of this review is on the contribution of medical imaging in lymphoma patients treated with CAR T-cells.

## Response Rates

CD19 CAR T-cell therapy has a high overall response rate with complete responses in up to 90% in adult and pediatric acute lymphoblastic leukemia motivating the initiation of hundreds of CAR T-cell clinical trials worldwide and the search for more efficient designs and new antigens (8, 9).

In clinical trials of aggressive lymphoma patients, complete response rates ranged from 40% to 59% (10). In indolent lymphoma, complete response rates were even higher (11, 12). Several factors, however, can limit long-term efficacy of CAR T-cells and lead to disease relapse (13). First, the delay to start CAR T-cell therapy may allow disease progression, with tumor volume increase, which stresses the importance of rapid and reliable manufacturing and bridging therapy. CAR T-cell function may also be decreased by the poor quality and low number of cells obtained from certain patients. In addition, treatment efficacy depends on the expression of the targeted antigen by the tumor cells. Therefore, tumor heterogeneity, mutations, down-regulation or loss of tumor antigen can decrease the recognition of tumors by CAR T-cells and subsequent therapeutic response. Moreover, CAR T-cells’ viability and efficacy can be impaired by suboptimal stimulation leading to T cells exhaustion and relapse (14).

In addition to poor effector to target ratio in presence of a high tumor burden (15), antigen positive relapse can also occur as a result of tumor cells resistance to CAR T-cells (14) or immunosuppressive tumor microenvironment (16) inducing CAR T-cell dysfunction.

## Baseline Biomarkers Predicting Response and Outcome

Biomarkers that predict short survival are critical for close monitoring during bridging therapy given that the manufacturing time may allow disease progression. Biomarkers that predict durable response at baseline may help identify patients more likely to benefit from this strategy. Biomarkers that indicate treatment failure at the 1-month milestone will identify patients who might benefit from early intervention such as therapies that reinvigorate CAR T-cells or a second infusion of CAR T-cells.

### Prognostic Value of Response Under Bridging Chemotherapy

In 72 patients with relapsed/refractory diffuse large B-cell lymphoma who received CAR T-cells, Tordo et al. measured the kinetics of tumor bulk during bridging therapy determined by the evolution of Total Metabolic Tumor Volume, Total Lesion Glycolysis or SUVmax. They demonstrated that biomarkers derived from the analysis of the kinetics of theses parameters during bridging therapy before lymphodepletion are better predictors of progression-free survival than baseline biomarkers (17). Thus, patients with satisfactory disease control before lymphodepletion had an overall longer progression-free survival.

### Tumor Volume on CT-Scan

On CT-scan, tumor volume is typically estimated using the sum of the product of perpendicular diameters of measurable target tumor lesions. In the first series reporting the efficacy of CAR T-cell in lymphoma, there was only a non-significant trend for the predictive and prognostic impact of tumor bulk assessed by morphological imaging. Schuster et al. found that the median sum of the product of perpendicular diameters was 20 cm2 (range 3-100) in responding patients and 30 cm2 (range 3-157) in non-responding patients (18). A recently published report on the TRANSCEND NHL01 trial also found a trend towards worse outcomes in patients with greater sum of the product of perpendicular diameters, with an objective response rate of 76.8% in patients with the sum of the product of perpendicular diameters <50 cm2 and 61.4% in patients with ≥50 cm2 (19). Likewise, Neelapu et al. found that patients with bulky disease (>10 cm) had an objective response rate of 71% (95CI: 0.44-0.90) compared with 85% (95CI: 0.75-0.91) for patients without bulky disease (20). More recently, an analysis in the same cohort found an association between tumor burden evaluated by the sum of the products of diameters of target lesions and durable response, however this parameter had limited sensitivity and specificity, which may be due to the fact that it does not take into account total tumor burden (15). Expansion of CAR T-cells in the blood was also predictive of response, along with markers of inflammation such as IL-6 and CRP. Interestingly, in this study the best predictor of durable response was the peak CAR T-cell levels in the blood normalized to pretreatment tumor burden. Durable responders had a higher peak CAR T-cell to tumor burden ratio than non-responders or responding patients relapsing within one year.

### Tumor Volume on [18F]-FDG PET-Scan

[18F]-FDG PET is a routine, standard of care imaging study that estimates tumor glucose consumption. [18F]-FDG PET is preferred for the staging and restaging of FDG-avid lymphomas because it outperforms CT scans in these diseases (21). Additionally, the overall metabolic tumor volume or Total Metabolic Tumor Volume (i.e, metabolically active tumor volume with significantly increased glucose metabolism) assessed on [18F]-FDG PET has good prognostic value. For instance, higher tumor volume in aggressive lymphomas before initiating first-line chemotherapy predicts shorter progression-free and overall survival. Therefore, tumor volume along with other parameters such as International Prognostic Index, Eastern Cooperative Oncology Group performance status and cell of origin could improve patient risk stratification (22, 23). Several groups have explored the predictive/prognostic value of metabolic tumor volume in patients undergoing CAR T-cell therapy and preliminary results suggest that Total Metabolic Tumor Volume is a relevant imaging biomarker.

In a study done on a small cohort (n=19) of patients with non-Hodgkin lymphomas, with a best overall response rate of 79%, the median Total Metabolic Tumor Volume was 72 cm^3^. Lower tumor volume was observed in responding (58.1 cm^3^) than in non-responding patients (110.8 cm^3^), though this did not reach statistical significance (24). Likewise, overall survival was not significantly different in patients above and below the median (8.6 months vs 11.5 months). The absence of prognostic value might be due to the small size of this cohort. Nonetheless, the authors found that patients with more severe cytokine release syndrome (grade 3-4), had significantly higher Total Metabolic Tumor Volume than patients with no or mild cytokine release syndrome.

Dean et al. observed a stronger correlation between Total Metabolic Tumor Volume and outcome in a larger cohort of 96 patients with diffuse large B-cell lymphoma (25). In a sub-group of 48 patients the median Total Metabolic Tumor Volume (determined by a manual method) was 147 mL. Lower tumor volume was associated with prolonged overall and progression-free survival. This was validated in a second sub-group (n=48), where median Total Metabolic Tumor Volume was lower (72.8 mL), and in the entire study population. Lower tumor volume was also associated with higher overall and complete response rates. In a subgroup of 72 patients with “true baseline” PET (no bridging therapy, or PET performed after bridging chemotherapy) the same results were observed. In addition, high tumor volume was associated with more grade 3-4 cytokine release syndrome but not with neurotoxicity.

In another study (n=116 patients with aggressive non-Hodgkin lymphoma), extension of lymphoma measured by more than two involved extranodal sites both at times of enrollment (decision of CAR T-cells and before bridging therapy if applicable) and treatment, and high Total Metabolic Tumor Volume (superior to 80 mL) at the time of treatment were predictive of progression-free survival, overall survival, and early progression (occurring during the first month) after CAR T-cell treatment in patients with R/R diffuse large B-cell lymphoma. Of note, elevated CRP at time of CAR T-cell infusion was also associated with a worse outcome (but with a low odds ratio). Combining the number of extranodal sites>2 and high tumor volume (>80 mL) allowed to establish 3 prognostic groups with 0, 1 or 2 adverse parameters, more distinctly than the revised International Prognostic Index (26).

## On-Treatment Imaging Biomarkers: Measuring Response

### Learning Curve

Since CAR-T-cell therapy is relatively novel, there is a critical need to evaluate the reproducibility in assessing response since even expert radiologists will have to familiarize with the patterns of response to CAR T-cells. The optimal time point for follow-up and therapeutic evaluation is not known and on initial studies was determined empirically. Second, pseudoprogression may occur, as observed in other immunotherapies, but there are no definitive criteria to define it. Finally, immune response could generate atypical uptake linked to an inflammatory process, such as observed with immunotherapy (colitis, thyroiditis…) (27–29).

### Typical Patterns of Response and Progression on CT-Scan

In 101 patients with relapsed/refractory aggressive B-cell non-Hodgkin lymphoma enrolled in the ZUMA-1 study, axicabtagene ciloleucel (axi-cel) had an overall response rate of 83% and complete response rate of 58%. The median duration of response was 11 months. Ongoing long-term responses were seen in 39% of patients after a median follow up of 27.1 months (30). In a recent update, the three-year overall survival was 47% (31). Eleven out of 33 patients with partial responses at 1 month, and 11 of 24 patients with stable disease at 1 month, subsequently attained a complete response without any additional therapy (30). In these cases of responses improving over time, complete resolution of FDG-avid lesions after CAR T-cell therapy may take up to 9-12 months and anecdotally even longer. Additionally, a complete response and partial response at the 3-month milestone were associated with similar progression-free survival, further highlighting the complexity in using early imaging findings for prognostic purposes.

Real-world evidence using commercial CAR T-cells, including patients with comorbidities found similar results with overall response rate of 82% and complete response rate of 64%, with estimated 12-months PFS of 47% and OS of 68% (32). Among the patients with a partial response at 1 month 32% achieved complete response at 3 months and only 1 out of 14 patients with stable disease achieved a complete response at 3 months.

### Typical Patterns of Response and Progression on [18F]-FDG PET

Shah et al. reported a case series of 7 patients with aggressive and indolent non-Hodgkin lymphoma treated with CAR T-cells, evaluated with [18F]-FDG PET (33). Three of these patients (all with follicular lymphoma) had a complete metabolic response at 1 month and remained disease-free at 2 years. Two patients with a partial metabolic response experienced later progression, at 3- and 6-months post-infusion respectively, and two patients had progressive disease as soon as 1 month post infusion. At this time-point, no Cytokine Release Syndrome-related metabolic activity impaired FDG PET interpretation.

A recent retrospective report on 10 patients with aggressive lymphoma, underlined the importance of early evaluation of therapeutic efficacy, with all patients with a partial or metabolic response at 3 months having shown metabolic response at 1 month, and only one patient with unfavorable outcome experiencing early metabolic response (34).

**Figure 1:**
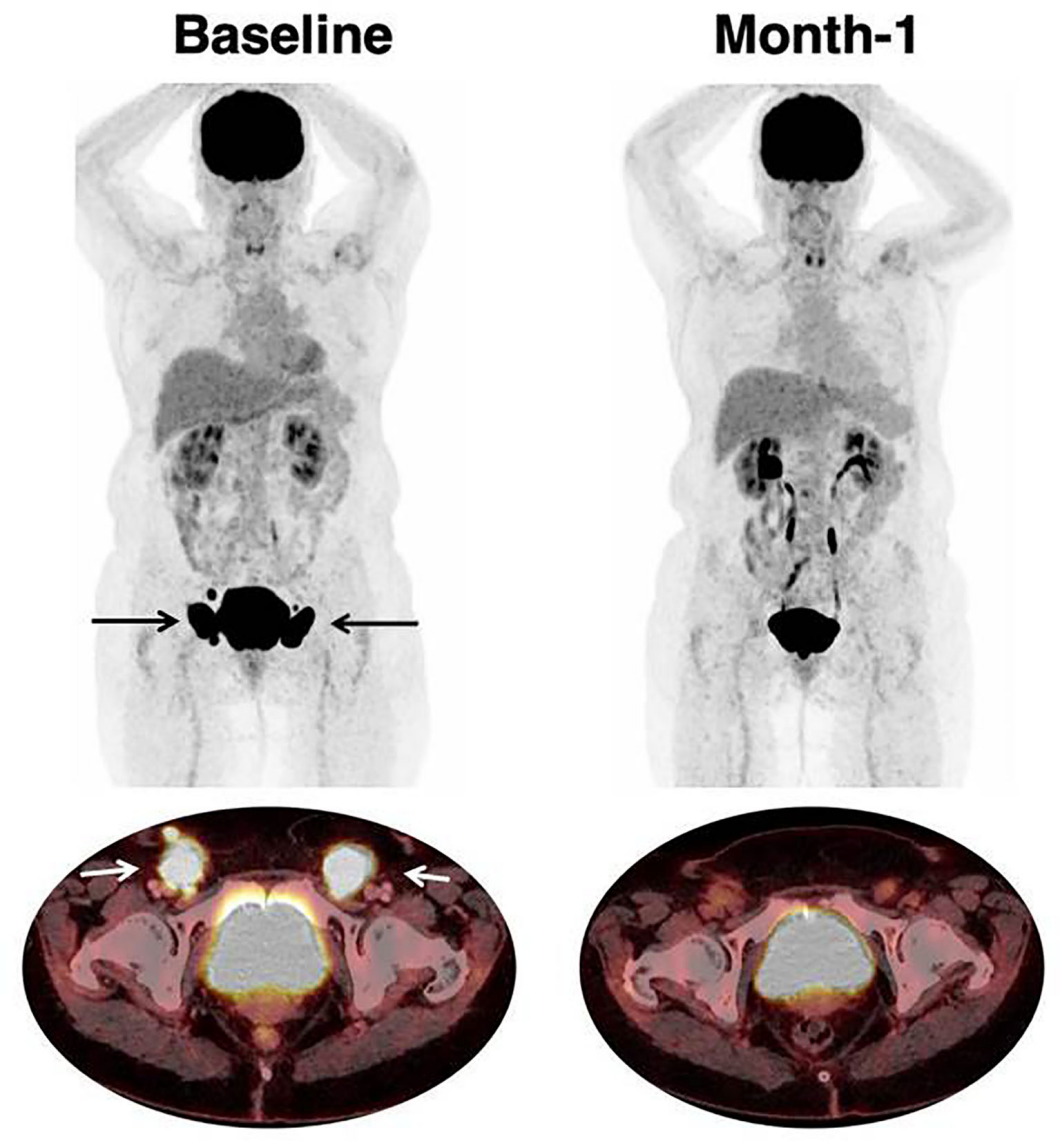
Response to CAR T-cell therapy. 66 year-old patient with past medical history of follicular lymphoma. The patient relapsed with DLBCL, treated with two lines of prior chemotherapy. Baseline imaging showed a low tumor volume (TMTV was 47 mm^3^) which is typically associated with favorable outcome and response to CAR T-cell therapy. Inguinal lymphadenopathies are indicated with black (on Maximum Intensity Projection) and white arrows (on axial fusion image). Follow-up imaging showed a partial response on CT-scan with residual disease. [18F]-FDG PET reclassified this patient as a complete metabolic response which persisted at month-6.

**Figure 2:**
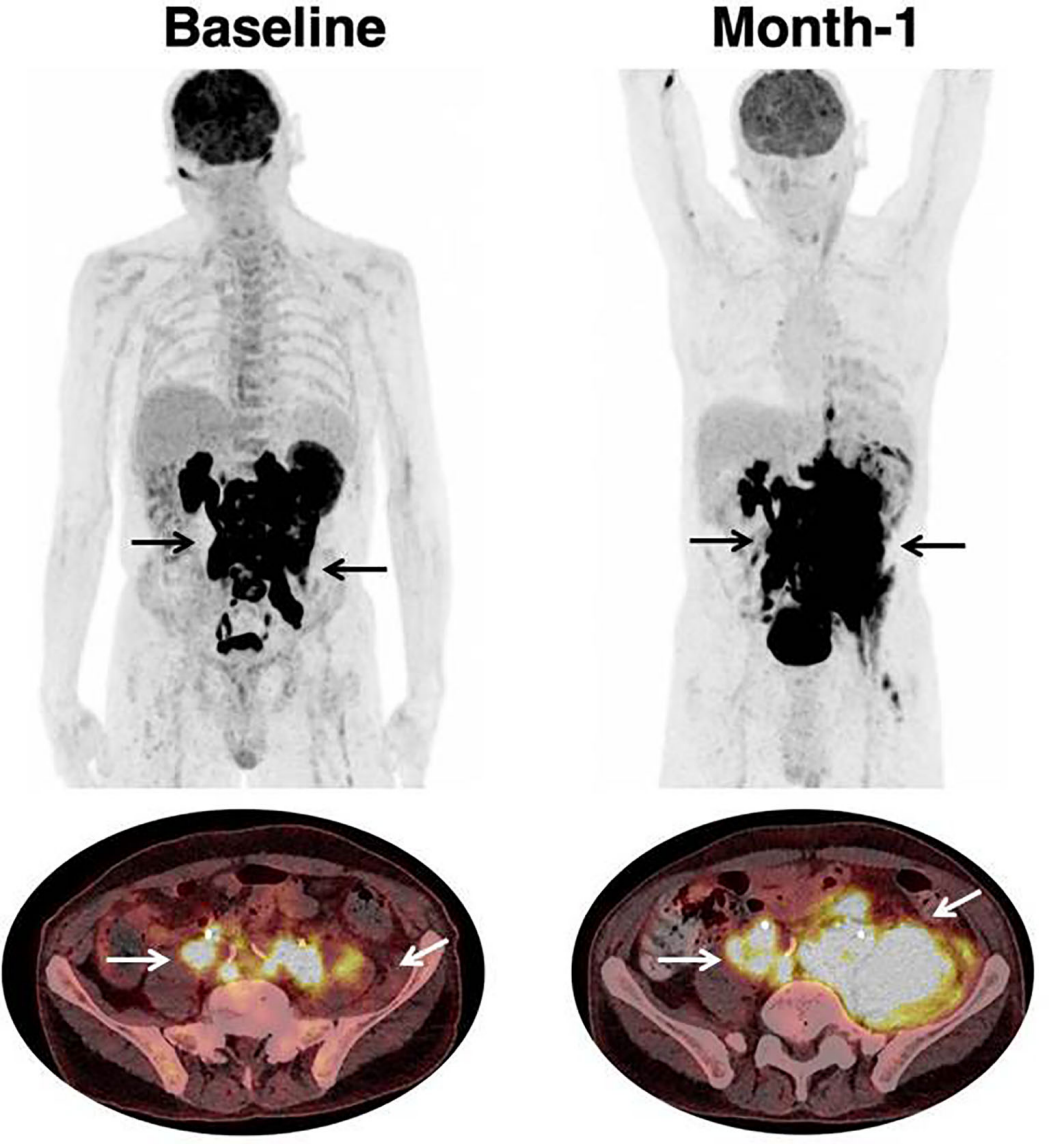
Progression in a patient treated with CAR T-cell therapy. 68 year-old patient with past medical history of DLBCL diagnosed one year prior to treatment initiation. Patient had Stage IV disease, with rearrangement of the MYC and BCL6 genes, and treated with two prior lines of chemotherapy. Black (on Maximum Intensity Projection) and white arrows (on axial fusion images) show infradiaphragmatic lymphadenopathies, with muscular infiltration. Baseline imaging showed high tumor volume which is typically associated with unfavorable outcome and lower response rate to CAR T-cell therapy. Follow-up imaging showed a progression on CT-scan as well as on [18F]-FDG PET. At month-1, there were new lesions as well as an increase in tumor volume. The prognosis was poor; hence salvage treatment and later best supportive care were initiated. Patient died at month-2.

Examples of therapeutic response assessment with FDG PET are presented in **Figures 1** and **2**.

### Atypical Patterns of Response and Progression

Wang et al. reported 3 cases of pseudoprogression that may cause local complications, due to compression of adjacent organs for example. Compared with other immunotherapies, pseudoprogression was very early, occurring as soon as 4 to 5 days after CAR T-cell infusion (24). More studies will be needed to better understand and describe the different patterns of response after CAR T-cell therapy.

## Toxicity

CAR T-cell is associated with a wide range of toxicities. Across studies, all patients experience at least one adverse event. On the most serious side of the spectrum are high grades Cytokine Release Syndrome (CRS) and Immune effector cell-associated Neurotoxicities Syndrome (ICANS). CRS is a systemic inflammatory response characterized among others by fever, hypotension, hypoxia and potential multiple organ failure whereas ICANS is characterized by various neurologic symptoms ranging from confusion and tremor to aphasia, dysgraphia, seizures or coma. These generally occur in the first weeks after CAR T-cell infusion as a results of high levels of cytokines not only produced by CAR T-cells but also by their activation of myeloid cells (3, 35). Pyrexia, fatigue, cytopenias and infections are also frequent after CAR T-cell therapy.

In the ZUMA-1 study, axi-cel infusion was associated with 11% incidence of grade 3 or higher CRS and 32% of ICANS grade 3 or more, with similar results in the real-world data described by Nastoupil et al. (30, 32, 36). In the JULIET study, CRS occurred in 58% patients, with grade 3 or higher CRS in 22%, while neurologic events were observed in 21% of patients, and grade 3 or higher in 12% (37). As mentioned above, several analyses showed that higher baseline tumor burden was associated with severe CRS (24, 25).

For neurotoxicity, along with abnormalities on electroencephalogram and transcranial Doppler ultrasound, dedicated brain FDG PET can contribute to diagnosis (the main observed abnormalities being cortical hypometabolism) and follow-up (38). Structural abnormalities are usually absent, but CT and MRI may identify concomitant events such as ischemic stroke or subarachnoid hemorrhage (38). However, there is a lack of prospectively collected data on the subject. Additionally, a recent study suggested that a higher FDG avidity of lymphoma, evaluated by SUVmax, was associated with more neurotoxicity (34). If confirmed in larger studies, this could also be of importance for patient management after CAR T-cell infusion. The mainstay of treatment of these toxicities is steroids, vasopressors and tocilizumab treatments.

## Challenges and Perspectives

There are several challenges to improve implementation of CAR-T-cells in routine clinical practice and the outcome of patients receiving this innovative treatment. These challenges include technical challenges (CAR-T-cell development, manufacturing), standardization of clinical trial results to facilitate the comparison (protocols, pre-conditioning of patients, CAR-T-cell formulation, quality and persistence), and identifying robust tools to optimize treatment decision (4, 7). Among these tools, imaging techniques may play a critical role. The role and use of medical imaging techniques remain to be defined but results presented above suggest that imaging will be a pivotal tool to guide treatment decisions.

Beyond Total Metabolic Tumor Volume, recent data suggest that lesion dissemination assessed on PET/CT by means of the largest distance between two lesions (normalized with the body surface area), contributes to assess the spread of the disease, and has a prognostic value, independently of Total Metabolic Tumor Volume in a cohort of first-line chemotherapy diffuse large B-cell lymphoma (39). Radiomics could further contribute to extract clinically meaningful data from medical images. Recent findings from a wide range of solid tumor types suggest that a signature combining a limited subset of pretreatment (40) or on-treatment (41, 42) imaging biomarkers are able to help to identify patients who might benefit from early intervention. Among these quantitative imaging biomarkers, several have been shown to predict responses to immunotherapies with immune checkpoint blockers such as increased tumor volume, increased tumor glucose metabolism, tumor organotropism in visceral tissues, and lower skeletal muscle index. These are all associated with unfavorable outcomes (43–47).

Hence, it is very likely that similar technologies will be applicable to CAR T-cells and that computational models will be applied to data from CT or PET/CT scans to predict outcome, while accounting for technical variability between machines and centers. These tools eliminate the bias of investigator assessment and multicenter variability, allowing their implementation in large multisite trials. Artificial intelligence could be used to combine previously cited biomarkers to build robust prognostic/predictive models. One challenge is that building robust models using artificial intelligence requires creating large datasets, hence the need to aggregate data from multiple institutions to avoid overfitting (48, 49). Eventually, deep learning could contribute to determine radiomics signature correlated with survival.

## Conclusion

The role of medical imaging, and PET/CT in particular, in lymphoma patients treated with CAR T-cells is twofold. First, the pre-infusion Total Metabolic Tumor Volume seems promising for its prognostic value, and should probably be associated with biological parameters, such as CRP at time of lymphodepletion (26, 50). Some data also suggest that high tumor volume could be correlated with more severe cytokine release syndrome, with possible direct impact on patient management and monitoring. Second, the evaluation of response with CAR T-cell is an ongoing challenge, with more data needed, especially on the possibility of pseudoprogression, slow or late responses as well as the timing of relapses. Beyond FDG, a better knowledge and understanding of imaging data could contribute to detect and treat toxicities timely (51) and further tailor the therapeutic strategy, with the use of next-generation CAR T-cells, combination therapeutics especially in patients with high tumor burden and potentially rapid implementation of salvage therapies in case of relapse such as new CAR T-cell infusion (targeting the same or other antigens), immunomodulatory agents or radiation therapy (52).

## Author Contributions

LV, DJ, and LD designed the mini review, drafted the work, and approved the content. RB, SK, RR, and LS revised it critically for important intellectual content and approved the content. All authors contributed to the article and approved the submitted version.

## Conflict of Interest

LS is a paid advisory board member for Roche and Novartis, and reports receiving commercial research grants from Merck and Boehringer Ingelheim. RR has consulting or advisory role: Atara Biotherapeutics, Novartis, Magenta Therapeutics, Bristol-Myers Squibb, Gilead Sciences, and received research Funding from Atara Biotherapeutics, Incyte, Pharmacyclics, Shire, Immatics, Takeda, Gilead Sciences, Precision Biosciences, Astellas Pharma. RB received honoraria from Novartis and Gilead.

The remaining authors declare that the research was conducted in the absence of any commercial or financial relationships that could be construed as a potential conflict of interest.
